# Inhibition of Vascular Endothelial Cadherin Cleavage Prevents Elastic Fiber Alterations and Atherosclerosis Induced by Intermittent Hypoxia in the Mouse Aorta

**DOI:** 10.3390/ijms23137012

**Published:** 2022-06-24

**Authors:** Olfa Harki, Sophie Bouyon, Marine Sallé, Alejandro Arco-Hierves, Emeline Lemarié, Alexandra Demory, Carole Chirica, Isabelle Vilgrain, Jean-Louis Pépin, Gilles Faury, Anne Briançon-Marjollet

**Affiliations:** 1 Université Grenoble Alpes, INSERM U1300, CHU Grenoble Alpes, Laboratoire HP2, 38042 Grenoble, France; olfa.harki@univ-grenoble-alpes.fr (O.H.); sophie.bouyon@univ-grenoble-alpes.fr (S.B.); marinesalle@laposte.net (M.S.); alejandro.arco-hierves@etu.univ-grenoble-alpes.fr (A.A.-H.); emeline.lemarie@univ-grenoble-alpes.fr (E.L.); alexandrademory2020@gmail.com (A.D.); jpepin@chu-grenoble.fr (J.-L.P.); anne.briancon@univ-grenoble-alpes.fr (A.B.-M.); 2 Unité Biochimie Immunoanalyse, Service de Biochimie SB2TE, CHU Grenoble Alpes, 38000 Grenoble, France; cchirica@chu-grenoble.fr; 3 Université Grenoble Alpes, INSERM U1292, CEA, 38042 Grenoble, France; isabelle.vilgrain@cea.fr

**Keywords:** obstructive sleep apnea syndrome, intermittent hypoxia, endothelial dysfunction, atherosclerosis, VE-cadherin, elastic fiber fragmentation

## Abstract

Intermittent hypoxia (IH), the major feature of obstructive sleep apnea syndrome (OSAS), induces atherosclerosis and elastic fiber alterations. VE-cadherin cleavage is increased in OSAS patients and in an IH-cellular model. It is mediated by HIF-1 and Src-tyr-kinases pathways and results in endothelial hyperpermeability. Our aim was to determine whether blocking VE-cadherin cleavage in vivo could be an efficient strategy to inhibit deleterious IH-induced vascular remodeling, elastic fiber defects and atherogenesis. VE-cadherin regulation, aortic remodeling and atherosclerosis were studied in IH-exposed C57Bl/6J or ApoE-/-mice treated or not with Src-tyr-kinases inhibitors (Saracatinib/Pazopanib) or a HIF-1 inhibitor (Acriflavine). Human aortic endothelial cells were exposed to IH and treated with the same inhibitors. LDL and the monocytes transendothelium passage were measured. In vitro, IH increased transendothelium LDL and monocytes passage, and the tested inhibitors prevented these effects. In mice, IH decreased VE-cadherin expression and increased plasmatic sVE level, intima-media thickness, elastic fiber alterations and atherosclerosis, while the inhibitors prevented these in vivo effects. In vivo inhibition of HIF-1 and Src tyr kinase pathways were associated with the prevention of IH-induced elastic fiber/lamella degradation and atherogenesis, which suggests that VE-cadherin could be an important target to limit atherogenesis and progression of arterial stiffness in OSAS.

## 1. Introduction

Cardiovascular diseases (CVDs) are an important public health issue, as the leading cause of morbidity and mortality, especially in elderly people [1]. Among risk factors for CVDs, obstructive sleep apnea syndrome (OSAS), characterized by repeated apneas and hypopneas during sleep due to upper airway obstructions, is one of the most frequent chronic diseases, affecting nearly one billion persons worldwide [2]. OSAS is associated with an increased risk of hypertension, atherosclerosis, stroke and myocardial infarction, and thus represents a high burden for patients and society [3]. The first-line treatment of OSAS is Continuous Positive Airway Pressure (CPAP) for reopening and stabilizing the upper airway. CPAP improves both symptoms and quality of life of OSAS patients, although it has a limited impact on late cardiovascular events, such as cardiac infarct or stroke [4]. Thus, the modern conception of OSAS treatment is to combine CPAP with other therapeutic approaches, which requires the identification of the mechanisms leading to the cardiovascular conditions of OSAS patients. 

OSAS is characterized by intermittent hypoxia (IH), which is the main feature leading to cardiovascular complications. The link between OSAS/IH and the structural and functional impairment of vascular endothelium has been well established in both patients and animal models. Indeed, IH induces various mechanisms, such as elevation of oxidative stress, activation of Hypoxia-Inducible Factor-1 (HIF-1) and its target genes, such as VEGF [5,6], low grade chronic inflammation and sympathetic activation that, in turn, lead to endothelial dysfunction, hypertension, vascular wall thickening and degradation of the elastic fibers [7,8,9,10,11] and atherosclerosis [3,12,13]. 

The integrity of the vascular endothelium is regulated by, among other proteins, vascular endothelial cadherin (= VE-cadherin = CD144), the major protein of the endothelial adherens junctions. In different situations, VE-cadherin undergoes enzymatic cleavage of its extracellular domain, which then becomes soluble (sVE) and detectable in the blood of patients affected by various cardiovascular comorbidities. This has been reported for OSAS, rheumatoid arthritis and some cancers, suggesting its potential as a biomarker for cardiovascular risk in these pathologies [14,15,16]. VE-cadherin cleavage is activated after phosphorylation of one of its tyrosine residues by the tyrosine kinases Src in response to endothelial cell stimulation by circulating factors such as VEGF, which activates the VEGF receptor tyrosine kinases and TNF-α [15,16]. We recently demonstrated that IH induces the VE-cadherin cleavage process by pathways that involve reactive oxygen species (ROS), HIF-1, the VEGF receptor (VEGFR) and tyrosine kinases, particularly Src-kinases. This cascade of activation is associated with elevated endothelial permeability in vitro [14]. Actually, endothelial hyperpermeability, a crucial and early step in atherogenesis, could facilitate lipid flux and inflammatory molecule/cell migration across the endothelium [17]. However, the disruption of endothelial integrity and increase in endothelium permeability has only been sparsely studied in OSAS patients and experimental models of IH, such as in rodents [18,19] and cell cultures [14,19,20,21], and the contribution of hyperpermeability to early atherogenesis induced by IH is not well described. We therefore hypothesized that IH could increase in vivo endothelial permeability through its favoring action on HIF-1/VEGFR/Src-mediated VE-cadherin cleavage, thereby enhancing transendothelium flux of proatherogenic molecules/cells and leading to early arterial wall alterations, including elastic fiber degradation and atherosclerotic lesions. 

Thus, our objective was to use both cellular and rodent models to investigate whether (i) IH-induced VE-cadherin cleavage and endothelial permeability participate in deleterious IH-induced arterial remodeling featuring elastic fiber degradation and atherogenesis, and (ii) these IH effects could be inhibited or abolished by blocking VE-cadherin cleavage with pharmacological inhibitors.

## 2. Results

### 2.1. Intermittent Hypoxia Increases Transendothelium Passage of LDL and Monocytes Migration In Vitro and Blocking the Pathways Involved in VE-Cadherin Cleavage Prevents these Effects 

**Figure 1:**
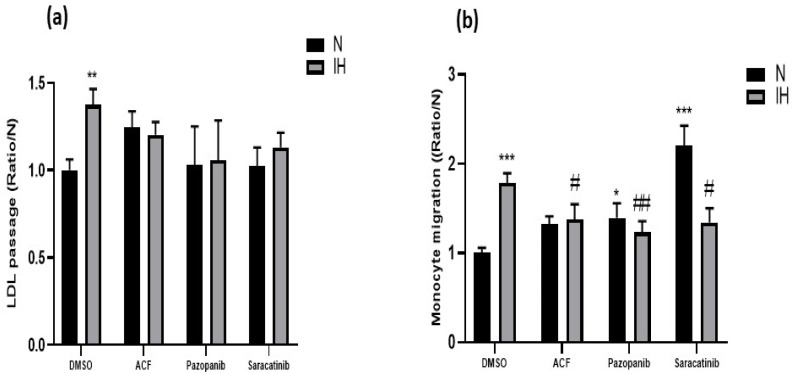
IH-induced increases in transendothelium passage of LDL and monocytes in vitro are prevented by inhibitors of VE-cadherin cleavage. (**a**) Inhibiting HIF-1 by acriflavine, Src-kinases by saracatinib and VEGF receptor tyrosine kinases by pazopanib, prevented the IH-induced increase in transendothelium passage of LDL (2-way ANOVA, ** *p* < 0.01 vs. N + DMSO, *n*= 28 for DMSO, *n* = 8–12). (**b**) Inhibiting HIF-1 by acriflavine, Src-kinases by saracatinib and VEGF receptor tyrosine kinases by pazopanib prevented the IH-induced transendothelium migration of monocytes (2-way ANOVA, * *p* < 0.05 and *** *p* < 0.001 vs. N + DMSO, # *p* < 0.05 and ## *p* < 0.01 vs. IH + DMSO, *n* = 10–11). Values are mean ± SEM. Control experiments: DMSO (vehicle of the inhibitors). N: normoxia, IH: intermittent hypoxia.

Using a transwell system, we showed that IH induced a significant increase in LDL passage (+37%, Figure 1a, bars: DMSO) and monocyte migration (+78%, Figure 1b, bars: DMSO) through an endothelial cell monolayer, compared to the situation under normoxia (N). 

We then used the HIF-1, VEGF receptor kinase and Src-kinase inhibitors acriflavine (ACF), pazopanib and saracatinib, respectively, that were previously demonstrated to block IH-induced VE-cadherin cleavage and endothelial permeability [14]. The three inhibitors abolished both the elevation of LDL passage (Figure 1a) and monocyte migration (Figure 1b) induced by IH.

### 2.2. Intermittent Hypoxia Induces VE-Cadherin Cleavage In Vivo in C57BL/6J Mice, and Inhibition of HIF-1, VEGRF Tyr-Kinases and Src-Kinases Prevents this Effect 

**Figure 2:**
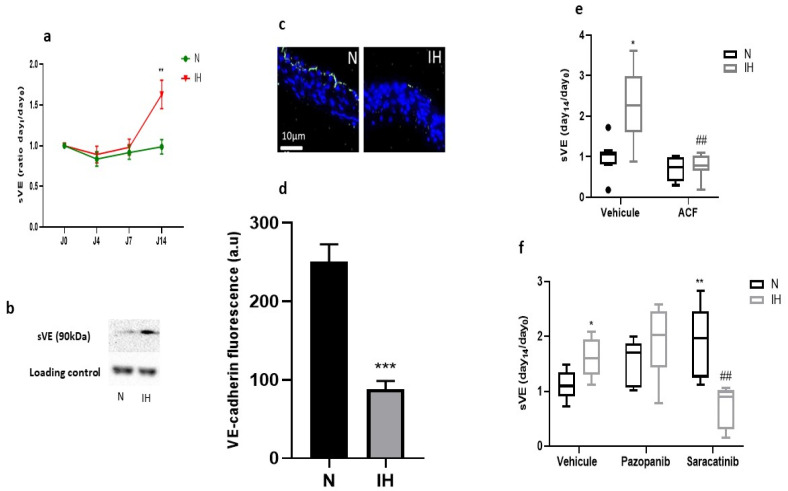
Intermittent hypoxia impairs VE-cadherin integrity in vivo in C57BL/6J mice, which is prevented by inhibitors of VE-cadherin cleavage. (**a**) IH increased sVE level in the plasma of C57BL/6J mice treated with vehicle (DMSO) after 14 days of exposure to IH, semi-quantitatively measured by Western blotting (N vs. IH at day 14, ** *p* < 0.01, Welch *t*-test, *n* = 21–22, values are mean ± SEM). (**b**) A representative example of Western blot showing a more intense sVE-band in the plasma of mice exposed to IH compared to those exposed to normoxia for 14 days (the immunoglobulin band is used as a loading control for normalization). (**c**) A representative example showing a more intense VE-cadherin fluorescence on the endothelium in N not in IH. Arrows indicate the VE-cadherin fluorescence in green in the endothelium, and the blue staining corresponds to nuclei stained with DAPI. (**d**) IH reduced VE-cadherin expression in the endothelium of C57BL/6J mice after 14 days of exposure to IH (N vs. IH at day 14, *** *p* < 0.001, Welch *t*-test, *n* = 15–16, values are mean ± SEM). (**e**) Inhibiting HIF-1 (by ACF) prevented the IH-induced elevation of sVE level in the plasma of C57BL/6J mice exposed for 14 days to IH or N (Kruskal-Wallis test, * *p* < 0.05 vs. N + vehicle, ## *p* < 0.01 vs. IH + vehicle, *n* = 6–8, values are median + interquartile range). (**f**) Inhibiting VEGFR tyr-kinases (by pazopanib) or src-kinases (by saracatinib) prevented the IH-induced elevation of sVE level in the plasma of C57BL/6J mice exposed for 14 days to IH or N (Kruskal-Wallis test, * *p* < 0.05 and ** *p* < 0.01 vs. N + vehicle, ## *p* < 0.01 vs. IH + vehicle, *n* = 5–8, values are median + interquartile range). N: normoxia, IH: intermittent hypoxia.

In vivo, after verification of the induction of HIF-1 in the aortic wall by IH (Supplementary data S1), we showed that a 2-week exposure to IH was sufficient to stimulate VE-cadherin cleavage in C57BL/6J mice. Indeed, we observed a significant increase in sVE level in the plasma of IH-exposed mice (+65%) compared to N-exposed mice (Figure 2a,b). This effect was associated with a depletion, in the same proportion (−65%), of the expression of full-length VE-cadherin in the aortic endothelium, as detected by immunofluorescence (Figure 2c,d). We then found that acriflavine, pazopanib and saracatinib prevented the IH-induced elevation of VE-cadherin cleavage (Figure 2e,f). Surprisingly, after saracatinib treatment, plasma sVE level was increased in N-exposed animals and decreased below normal levels in IH-exposed animals compared to the values found in corresponding animals treated with vehicle alone (Figure 2f).

### 2.3. Inhibiting HIF-1, VEGRF Tyr-Kinases and Src-Kinases Prevents the Effect of Intermittent Hypoxia on Intima-Media Thickness and Elastic Lamella Integrity In Vivo in C57BL/6J Mice 

**Figure 3:**
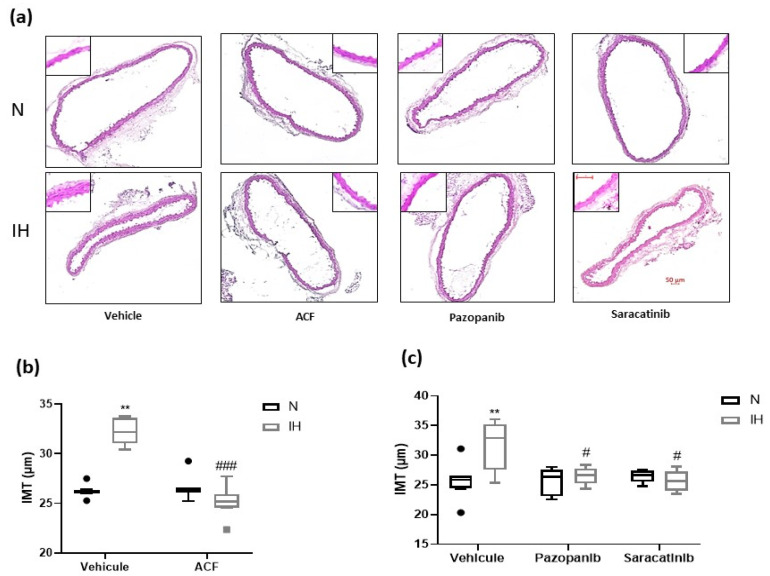
The effect of the inhibition of VE-cadherin cleavage pathways on intima-media thickness in C57BL/6J mice. (**a**) Representative images of aortic cross-sections stained with Hematoxylin-Eosin. (**b**) Inhibiting HIF-1 (by ACF) prevented the IH-induced elevation of IMT in C57BL/6J mice exposed to IH for 14 days, compared to N (Kruskal-Wallis test, ** *p* < 0.01 vs. N + vehicle, ### *p* < 0.001 vs. IH + vehicle, *n* = 7–8). (**c**) Inhibiting VEGFR tyr-kinases (by pazopanib) and src-kinases (by saracatinib) prevented the IH-induced elevation of IMT in C57BL/6J mice exposed 14 days to IH, compared to N (Kruskal-Wallis test, ** *p* < 0.01 vs. N + vehicle, # *p* < 0.05 vs. IH + vehicle, *n* = 5–8). (N: normoxia, IH: intermittent hypoxia. Values are median + Interquartile range. Scale bars = 50 µm.

**Figure 4:**
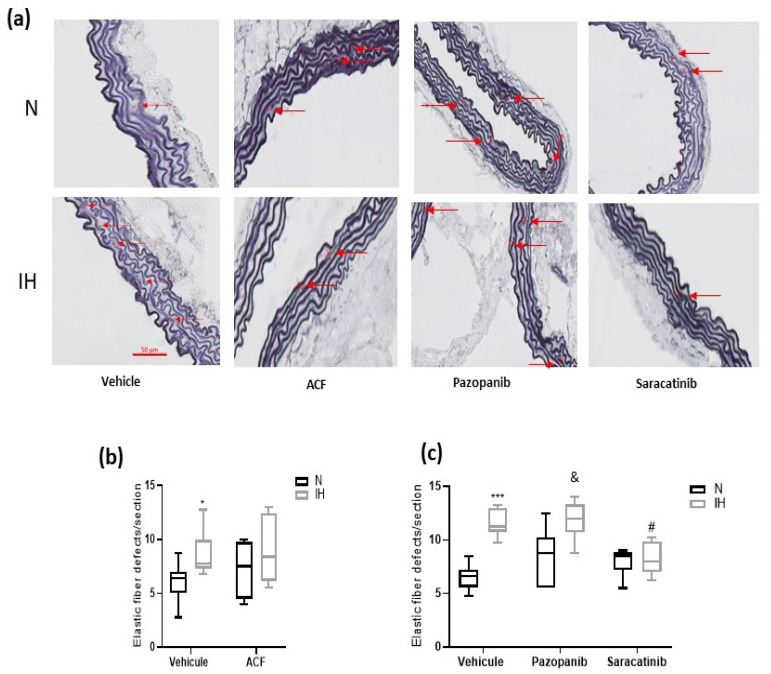

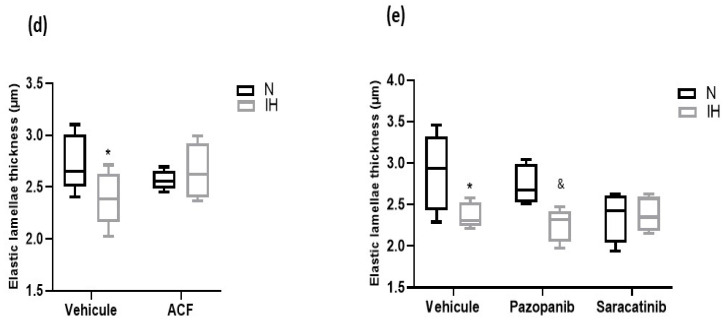
The effect of the inhibition of VE-cadherin cleavage pathways on aortic elastic lamellae in C57BL/6J mice. (**a**) Representative images of Weigert-stained aorta cross-sections (red arrows indicate elastic lamella disruptions). (**b**) Inhibiting HIF-1 (by ACF) prevented the elevation of elastic lamella disruptions induced by IH (Kruskal-Wallis test, * *p* < 0.05 vs. N + vehicle, *n* = 7–8). (**c**) Inhibiting src-kinases (by saracatinib) prevented the elevation of elastic lamella disruptions induced by IH. Inhibiting VEGF receptor tyrosine kinases (by pazopanib) had no effect on the elevation of elastic lamella disruptions induced by IH (Kruskal-Wallis test, *** *p* < 0.001 vs. N + vehicle, # *p* < 0.05 vs. IH + vehicle, & *p* < 0.05 N + Pazopanib vs. IH + Pazopanib, *n* = 5–8). (**d**) Inhibiting HIF-1 (by ACF) prevented the IH-induced thinning of elastic lamellae (Kruskal-Wallis test, * *p* ≤ 0.05 vs. N + vehicle, *n* = 5). (**e**) Inhibiting src-kinases (by saracatinib) prevented the IH-induced thinning of elastic lamellae. Inhibiting VEGF receptor tyrosine kinases (by pazopanib) had no effect on the IH-induced thinning of elastic lamellae (Kruskal-Wallis test, * *p* ≤ 0.05 vs. N + vehicle, & *p* < 0.05 N + Pazopanib vs. IH + Pazopanib, *n* = 5). N: normoxia, IH: intermittent hypoxia. Mice were exposed to N or IH for 14 days. Values are median + Interquartile range. Scale bar = 50 µm.

To assess the IH-induced vascular remodeling and effect of the inhibitors, we first measured the intima–media thickness (IMT) in aortas of C57BL/6J mice exposed for 14 days to IH or N, treated by DMSO alone (control), acriflavine, pazopanib or saracatinib. We reproduced the known IH-induced elevation of IMT [12] and showed that all the inhibitors prevented this effect (Figure 3a,c). Second, we confirmed that IH alters the aortic elastic lamellae, and the elastic fibers (Figure 4a) in particular, by increasing the number of elastic lamella disruptions (Figure 4b,c) and reducing the thickness of the elastic lamellae (Figure 4d,e) without affecting the number of elastic lamellae (Supplementary data S5). This effect was totally prevented by acriflavine and saracatinib, while pazopanib had no effect (Figure 4). As a complement, we verified that IH decreased the weight and increased the hematocrit of the studied mice, while these effects were unchanged by the inhibitors (Supplementary data S2).

### 2.4. Inhibiting HIF-1, VEGRF Tyr-Kinases and Src-Kinases Prevents the Favoring Effect of Intermittent Hypoxia on Atherogenesis in ApoE-/- Mice

**Figure 5:**
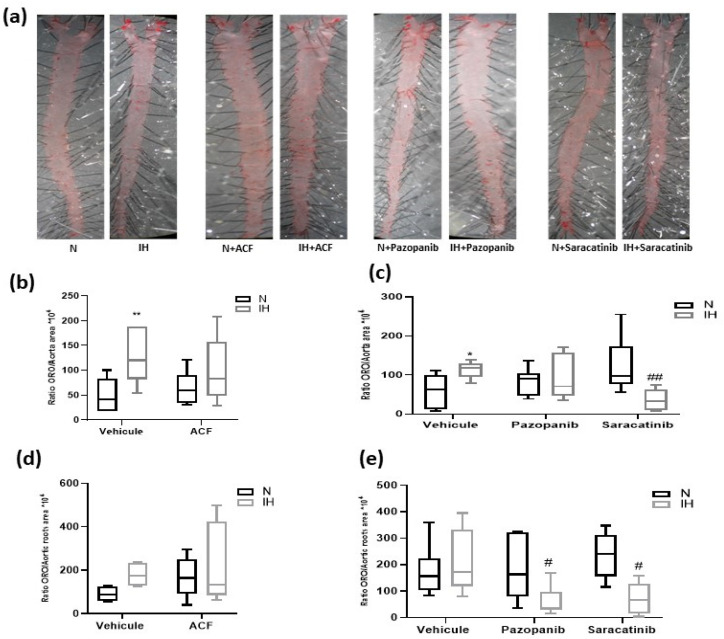
The impact of the inhibition of VE-cadherin cleavage pathways on atherosclerosis lesions in ApoE-/- mouse aortas. (**a**) Representative examples of ORO-stained entire aortas. (**b**) Inhibiting HIF-1 (by ACF) prevented atherosclerotic plaques in aortas of ApoE-/- mice exposed for 8 weeks to IH (Kruskal-Wallis test, ** *p* < 0.01 vs. N + vehicle, *n* = 5–8). (**c**) Inhibition of VEGFR tyr-kinases (by pazopanib) and src-kinases (by saracatinib) prevented the formation of atherosclerotic plaques in aortas of ApoE-/- mice exposed for 8 weeks to IH (Kruskal-Wallis test, * *p* < 0.05 vs. N + vehicle, ## *p* < 0.01 vs. IH + vehicle, *n* = 5–6). (**d**) Inhibiting HIF-1 (by ACF) tended to reduce atherosclerotic plaques in aortic roots of ApoE-/- mice exposed for 8 weeks to IH (Kruskal-Wallis test, *p* = 0.08 for N + vehicle vs. IH + vehicle, *n* = 4–8). (**e**) Inhibition of VEGFR tyr-kinases (by pazopanib) and src-kinases (by saracatinib) reduced the atherosclerotic plaque area in aortic roots of ApoE-/- mice exposed for 8 weeks to IH (Kruskal-Wallis test, # *p* < 0.05 vs. IH + vehicle, *n* = 5–6). N: normoxia, IH: intermittent hypoxia. Values are median + interquartile range.

We assessed the presence of atherosclerotic lesions by using Oil Red O staining of entire aortas and aortic roots from atherosclerosis-prone ApoE-/- mice, after 8 weeks of exposure to IH or N. We found that blocking VE-cadherin cleavage with the three inhibitors prevented the IH-induced enhancement of atherosclerotic plaque formation in aortas (Figure 5a–c). In the aortic roots, the pro-atherosclerotic effect of IH did not reach the significance level and trends were observed regarding the preventive effects of the inhibitors (Figure 5d,e). Finally, we verified that IH and the inhibitors had no effect on the animal weight (Supplementary data S3), while IH increased the hematocrit (Supplementary data S3). In the groups intraperitoneously injected with drugs (DMSO and acriflavine), IH induced only a trend towards elevation of hematocrit, the values not reaching the significance level. The inhibitors did not induce significant changes in plasma total-cholesterol levels (Supplementary data S4).

### 2.5. Inhibiting VEGRF Tyr-Kinases or Src-Kinases Abolishes the IH-Induced Increase in Arterial Blood Pressures in ApoE-/- Mice

**Figure 6:**
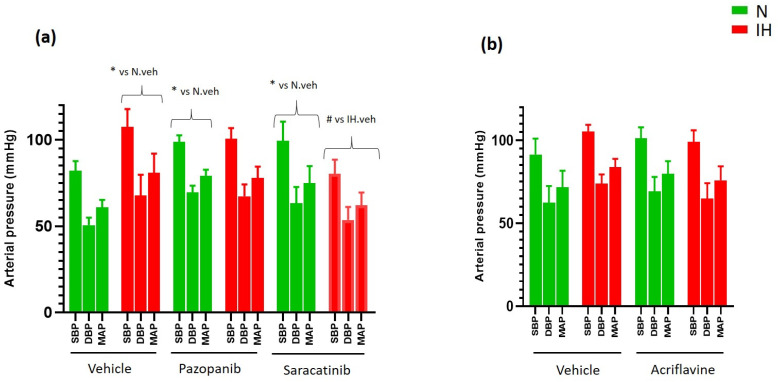
The impact of blocking VE-cadherin cleavage pathways on arterial blood pressures of ApoE-/- mice. (**a**) Inhibiting VEGFR tyr-kinases (by pazopanib) and src-kinases (by saracatinib) prevented the hypertensive effect of IH on arterial blood pressures in ApoE-/- mice exposed to IH for 8 weeks (3-way ANOVA, followed by a Fisher’s LSD test, * *p* < 0.05 vs. N + vehicle, # *p* < 0.05 vs. IH + vehicle, *n* = 5–8). (**b**) IH induced a trend towards elevation of blood pressure, and acriflavine tended to reduce this effect in ApoE-/- mice exposed to IH for 8 weeks (3-way ANOVA, *p* = 0.17 for the interaction IH × treatment, *n* = 5–8). Values are mean ± SEM. N: normoxia, IH: intermittent hypoxia. veh: vehicle. SBP = systolic blood pressure, DBP = diastolic blood pressure, MAP =mean blood pressure.

We measured arterial blood pressure by tail-cuff sphygmomanometry in ApoE-/- mice after 8 weeks of exposure to IH. We found that IH increased arterial systolic, diastolic and mean blood pressures, in accordance with the literature, and the VEGFR-tyrosine kinases and Src-tyrosine kinases inhibitors (pazopanib and saracatinib, respectively) prevented this effect (Figure 6a). In the groups intraperitoneously injected with drugs (DMSO or acriflavine), the hypertensive effect of IH did not reach the significance level and only trends were observed regarding the preventive effects of acriflavine (Figure 6b).

## 3. Discussion

Previous in vitro data suggested that IH induced VE-cadherin cleavage/sVE release and, in turn, increased endothelial permeability via the HIF-1/VEGFR tyr-kinases/src-kinases signaling pathways [14]. Here, we expanded these in vitro findings by in vivo physiopathology by inhibitor administration to mice. We first demonstrated that IH induced alterations of endothelial adherens junctions by cleavage of VE-cadherin and subsequent release of its soluble fragment (sVE), which were concomitant with hypertension, aortic wall thickening, elastic fiber/lamella alterations and atherogenesis. Second, we demonstrated that inhibition of HIF-1/VEGFR tyr-kinases/src-kinases signaling pathways prevented the occurrence of all these effects of IH, suggesting a link between VE-cadherin cleavage and these effects. 

The depletion of VE-cadherin from endothelial cell membranes is responsible for increased endothelial permeability [19,22,23], which could facilitate the infiltration of pro-atherogenic molecules or cells into the vascular wall. Supporting this hypothesis, we demonstrated, in vitro through an endothelial monolayer, an IH-induced increase in LDL passage (+37%) and monocyte migration (+78%), both being atherogenic mediators. This effect correlates with, and is probably due to, the IH-induced increase in endothelial permeability described previously [14], which could favor the paracellular passage of these molecules and cells [24,25] through the formation of intercellular gaps [14,20]. It has been demonstrated that these gaps are caused, at least in part, by disruption of the VE-cadherin function [20] due to the cleavage of its extracellular domain, sVE, by neutrophil surface-bound proteases [26]. As supporting evidence, plasma sVE level was found to be high in atherosclerosis in mice [27,28] and humans [29].

However, other mediators and pathways could also regulate vascular wall infiltration by LDL and monocytes. Actually, the endothelium permeability to LDL could be regulated by tight junction proteins, such as occludins and claudins [25], and by a transcellular route [25,30] through the cGMP/PKG/NF-kB signaling pathway [25]. Regarding leukocyte transendothelium migration, this could also involve the transcellular pathway [31] and JAM proteins [32,33]. Under IH, we previously demonstrated a ≈20% increase in the transendothelial passage of 40 kDa-dextran [14], a typical agent whose transport is conducted by a mostly paracellular route [34,35]. The higher transendothelium passage of LDL and monocytes under IH could thus be explained by the activation of a combination of paracellular and transcellular routes. Future studies will be required to explore and characterize the transcellular route in IH-induced endothelial permeability, which likely is another important contributor to IH-induced atherogenesis.

We then treated C57BL/6J mice with inhibitors of suggested VE-cadherin cleavage pathways, as described previously [14], and showed that an HIF-1 inhibitor (acriflavine), a VEGF receptor tyr-kinases inhibitor (pazopanib) and a Src-kinases inhibitor (saracatinib) abolished the IH-induced VE-cadherin cleavage in vivo in mice, reflected by a reduction of plasma sVE levels. This generalizes our previous results obtained in vitro [14] to in vivo physiopathology, showing that VE-cadherin cleavage could involve the HIF-1/VEGFR tyrosine-kinases/Src tyrosine kinase pathways. VEGF gene transcription was not modified by IH in our mice (data not shown); however, the significant impact of pazopanib could be linked to an upregulation of VEGF or VEGFR at the protein level, or of VEFR-induced signaling pathways in the cells exposed to IH. 

In C57BL/6J mice, IH generated the already known vascular remodeling (i.e., increased IMT) [12] and elastic fiber/lamella alteration [10,18,36] effects, which were prevented or inhibited by acriflavine, pazopanib and saracatinib, thereby suggesting the involvement of VE-cadherin-related endothelial permeability. This also opens a new possibility of a mechanistic explanation for the already demonstrated IH-induced arterial stiffening [7,8,9,11].

Since vascular remodeling is an early step of the atherogenesis process, we then analyzed atherosclerotic lesions induced by IH in ApoE-/- mice. We reproduced the known favoring effect of IH on the formation of atherosclerotic lesions [12] in entire aortas. The inhibition of VE-cadherin cleavage pathways by acriflavine, pazopanib and saracatinib was associated with a reduction of the overall atherosclerotic lesion size in the aortas, without any modification of plasmatic total cholesterol level. This suggests that IH-induced atherosclerosis is enhanced by the endothelial hyper-permeability mediated by VE-cadherin cleavage [14], which facilitates the infiltration of lipids and inflammatory cells in the arterial wall. This was confirmed in vitro by the abolition by inhibitors of the endothelium LDL passage and monocyte migration induced by IH. Our results are consistent with previous studies, out of the IH context, which demonstrated that the inhibition of VE-cadherin cleavage [37], tyrosine-kinases [37,38], src-kinases [39,40] and HIF-1 [41,42] protects against atherosclerosis in ApoE-/- mice. However, we could not exclude the contribution of other pathways activated by IH, such as cAMP/PKA/RhoA [19], inflammation [10,43], NF-kB/TNFα pathways [16,27], cysteinyl-leukotriene pathway [44] and cyclooxygenase pathway [45]. 

We finally assessed the effect of the inhibitors of VE-cadherin cleavage pathways on arterial pressures, since hypertension is a common cardiovascular complication of IH-induced endothelial dysfunction [12]. We first confirmed the elevation of the mouse blood pressure in response to 8 weeks of exposure to IH, an effect which was prevented by VEGF receptor tyr-kinase inhibitor (pazopanib) and a Src-kinase inhibitor (saracatinib). However, HIF-1 inhibition by acriflavine only tended to prevent this effect. 

Surprisingly, we noticed that in animals exposed to normoxia (N), saracatinib significantly increased plasma sVE and arterial pressures and induced a trend towards atherosclerosis lesion increase (Figure 5b,e, *p* = 0.07) compared to the normoxic group treated with the inhibitor vehicle only. These effects of saracatinib were not found under IH, and may be explained by the fact that in N, src-kinases are not active and have a conformation different from that of the active enzyme assumed to be present during IH [46]. When Src-kinases are in their inactive form (in N conditions), saracatinib could not efficiently bind to the ATP binding site, as it does with the active form of the enzyme, and therefore loses its specificity for the enzyme. This could lead to cardiovascular side effects similar to those of multitarget tyrosine kinases inhibitors (as dasatinib, nilotinib, and sunitinib), including a high risk of hyperlipidemia [47,48,49]. To a lesser extent, pazopanib tends to have effects resembling those of saracatinib, but the difference does not reach the significance level except for arterial pressure, the increase of which is a known side effect of pazopanib [50]. Moreover, anti-VEGF therapy is considered as a risk factor of increased IMT and thus of atherosclerosis [51]. This can be explained by the lack of specificity of the VEGF receptor tyr-kinases inhibitor, pazopanib, for the inactive form, and by the fact that this molecule targets multiple tyrosine kinases [49]. However, we did not observe a thickening of the aorta wall under blockade of VEGFR-related pathways, i.e., under pazopanib or saracatinib treatment.

Finally, a limit of our study is the absence of a direct and specific inhibitor of the cleavage of VE-cadherin. Y685F mice mutated on tyrosine 685, which is phosphorylated before cleavage, have been studied by two different teams [23,52]; however, they led to discordant results regarding endothelial permeability. Therefore, since there is no specific inhibitor of the cleavage of VE-cadherin, we used inhibitors of different molecular processes known [14] to be major contributors of this cleavage, such as VEGFR tyrosine kinases [15], src-kinases [16] and HIF-1, VEGF being a target gene of HIF-1 [3]. We demonstrated that the inhibition of VE-cadherin cleavage pathways by the three inhibitors used is associated with the prevention of IH-induced vascular remodeling, elastic fiber alteration atherosclerosis and hypertension in vivo, as well as endothelial permeability to LDL and monocytes in vitro. These findings provide new insights into IH-induced arterial dysfunctions and suggest a potential of VE-cadherin targeting for new anti-atherogenic, anti-hypertensive and anti-arterial stiffening therapeutic strategies to reduce cardiovascular complications associated with OSAS. 

## 4. Materials and Methods

### 4.1. Cell Culture

Cryopreserved primary human aortic endothelial cells (HAoEC, Thermo Fisher Scientific C0065C, Waltham, MA, USA) were used before their 8th passage. Cells were seeded at a density of 1.6 × 10^4^ cells/cm^2^ and cultured (at 37 °C, 5% CO_2_) in M200 medium (Thermo Scientific M200500, Waltham, MA, USA) supplemented with Large Vessel Endothelial Supplement LVES (Thermo Scientific A1460801, Waltham, MA, USA) and penicillin (1%)/streptomycin (1%) according to the supplier’s recommendations. 

Monocytes from the THP-1 human cell line were kindly provided by Dr. Fabienne Burger (Cardiology division, Geneva University Hospitals, Switzerland). They were maintained in RPMI medium (Thermo Scientific 61870, Waltham, MA, USA), supplemented with 25 mM HEPES, 10% fetal bovine serum (FBS), 1% penicillin/streptomycin and 0.1% fungizone, at 37 °C and 5% CO_2_.

All cell culture reagents were purchased from Gibco Thermo Fisher Scientific (Illkirch, France).

### 4.2. Intermittent Hypoxia Exposure of Cultured Cells 

Cells were exposed for 6 h to fast intermittent hypoxia (IH) cycles using a device that mimics the hypoxia-reoxygenation cycles of OSAS patients, as previously described [53]. The IH cycle included a 5-min normoxia (N) phase (16% O_2_) followed by a 5-min hypoxia phase (2% O_2_). 

### 4.3. Use of Inhibitors in In Vitro Experiments

In all experiments performed in cultured cells we used 1 μM saracatinib (AZD0530, a Src-kinase inhibitor), 0.5 μM acriflavine (an HIF-1 inhibitor), 5 μg/mL pazopanib (VEGF receptor tyrosine kinases inhibitor) solubilized in DMSO before addition to the cells. For control experiments, the same volume of DMSO alone was added to the cells to reach a final DMSO concentration of 0.1%. Acriflavine and pazopanib were from Sigma-Aldrich (Darmstadt, Germany, references A8126-25G and 9005-65-6, respectively), saracatinib was from AdooQ BioScience (Irvine, CA, USA, reference: A10108). The concentrations used were chosen according to the literature, and cell viability was verified after the treatment of HAEC cells as described previously [14].

### 4.4. In Vitro LDL Transendothelium Passage Assay 

HAoEC were seeded at a density of 5 × 10^4^ cells/well in transwells (permeable polystyrene membrane inserts, 6.5 mm diameter, 8 μm membrane pore size) from Corning (Sigma-Aldrich, Darmstadt, Germany, reference CLS3422-48EA) coated with 0.2 mg/mL type I collagen (Sigma-Aldrich, Darmstadt, Germany) in 50 µL/transwell M200 medium supplemented with LVES and penicillin/streptomycin. The lower chamber of the transwell was filled with 600 µL M200. After three days of incubation at 37 °C and 5% CO_2_, the transwells were transferred to 24-well plates with a gas-permeable bottom (Fluorocarbon Imaging plates, Zell-Kontakt, Hardenberg, Germany). Inhibitors or DMSO were diluted in M200 medium and added into the upper chamber of the transwells, while M200 medium was added to the lower chamber. The transwells were exposed to IH or N for 3 h. Then, 10 μg/mL fluorophore-labelled LDL (FL-LDL, Invitrogen, Waltham, MA, USA) was added into the upper chamber of the transwells and cells were re-incubated in N or IH in the device for an additional 3 h. Finally, media from the lower chamber of each transwell were collected and the quantity of FL-LDL was quantified using fluorescence detection (excitation: 515 nm, emission: 520 nm) according to the supplier’s recommendations. Values were normalized to the mean fluorescence value in normoxic control duplicates. 

### 4.5. In Vitro Monocyte Transendothelium Migration Assay 

HAoEC were seeded at a density of 5 × 10^4^ cells/well in transwells (permeable polystyrene membrane inserts, 6.5 mm diameter, 8 μm membrane pore size) from Corning (Sigma-Aldrich, Darmstadt, Germany, reference CLS3422-48EA) coated with 0.2 mg/mL type I collagen (Sigma-Aldrich, Darmstadt, Germany) in M200 medium supplemented with LVES and penicillin/streptomycin. After three days of incubation at 37 °C and 5% CO_2_, the transwells were transferred to 24-well plates with a gas-permeable bottom (Fluorocarbon Imaging plates, Zell-Kontakt, Hardenberg, Germany). The upper and lower chambers of the transwells were filled with 200 µL and 600 µL M200 medium, respectively, and exposed to IH or N for 2 h. Then, 6 × 10^5^ THP1 monocytes, diluted in 200 µL M200 medium, were added to the upper chamber of the transwells and 100 ng/mL of MCP1 (monocyte chemoattractant protein 1, R&D Systems, Minneapolis, MN, USA) was added to the lower chamber (inhibitors or DMSO were diluted in the same media). Transwells were re-incubated for an additional 4 h in the IH device. Then, monocytes which had migrated in the lower chamber of the transwells were concentrated in 50 µL M200 medium and counted using a Neubauer counting chamber. Values were normalized to the mean cell number in normoxic control transwells. 

### 4.6. Animals 

We used 80 wild type C57BL/6J mice (males, age = 12 weeks, weight = 26–27 g) purchased from Janvier Labs (Le Genest-Saint-Isle, France) to study the IH-induced vascular remodeling and effects of the inhibitors, and 61 ApoE-/- mice (C57BL/6J background, males, age = 12 ± 1 weeks, weight = 24–26 g) bred in our local animal facility to study the occurrence of IH-induced atherosclerotic lesions and effects of the inhibitors. All mice were fed on a standard diet and were weighed every week of exposure to IH. Animals were housed in a local facility (at 20–22 °C) and had free access to food and water.

### 4.7. Exposure to Intermittent Hypoxia

Wild type (WT) C57BL/6J mice were randomly exposed to IH or N for 2 weeks, while ApoE-/- mice were randomly exposed to IH or N for 8 weeks. The fraction of inspired oxygen (FiO_2_) in cages was monitored with a gas analyzer throughout the experiments. Intermittent hypoxia was generated by intermittent injection of low oxygen air in the cages, with 1-min cycles (30 s at 5% FiO_2_, 30 s at 21% FiO_2_ in the cages) repeated for 8 h/day (from 8:00 a.m. to 4:00 p.m.). Normoxic mice were exposed to the same air flow turbulences and noises as IH mice. The verification of the expected effect of IH was done by measuring HIF-1 expression in the arterial wall by immunofluorescence in aorta cryosections. As expected, IH-induced elevation of HIF-1 expression was observed (Supplementary data S1).

### 4.8. Treatment of Mice with VE-Cadherin Cleavage Inhibitors 

Wild type C57BL/6J mice were randomly distributed into 10 groups (G1 to G10) of eight mice per group. Four groups (G1 to G4) were treated by intraperitoneal injection (IP) and six groups (G5 to G10) were treated by gavage: G1 = N + vehicle by IP (Vehicle is PBS), G2 = HI + vehicle by IP (Vehicle is PBS), G3 = N + ACF 3 mg/kg/day by IP, G4 = HI + ACF 3 mg/kg/day by IP, G5= N + vehicle by gavage (vehicle is PBS + 0.5% HPMC (hydroxypropylmethylcellulose) + 0.1% Tween 80), G6 = HI + vehicle by gavage (vehicle is PBS + 0.5% HPMC (hydroxypropylmethylcellulose) + 0.1% Tween 80), G7 = N + Pazopanib 30 mg/kg/day by gavage, G8 = IH + Pazopanib 30 mg/kg/day by gavage, G9 = N + Saracatinib 20 mg/kg/day by gavage, G10 = IH + Saracatinib 20 mg/kg/day by gavage. In the results, groups treated by IP and gavage routes were represented on separate graphs. 

Similarly, ApoE-/- mice were randomly distributed into the same 10 groups, including 5–8 animals per group. For each inhibitor, ApoE-/- mice were treated during the 8 weeks of IH exposure (5 days per week) by using the same routes and doses as those used for wild-type C57BL/6J animals. 

In some groups, the final number of animals for which data were collected was lower than the initial number because a few animals died during the weeks of exposure to N or IH. 

### 4.9. Specimen Collection

At the end of the period of exposure to N or IH, the animals were anesthetized by an intraperitoneal injection of 100 mg/kg ketamine-10 mg/kg xylazine. The blood was collected by cardiac puncture in heparin tubes and hematocrit was measured (supplementary data S2 et S3). After a cardiac perfusion with PBS, aortas and carotids of C57BL/6J, aortic roots of APOE-/- mice were harvested and stored at −80 °C until analysis. The entire aortas of ApoE-/- mice were also harvested after cardiac perfusion with PBS and instantly fixed with 2% paraformaldehyde for 24 h, then saved in PBS at 4 °C until Oil Red-O staining.

### 4.10. sVE Semi-Quantitative Dosage by Western Blot

Plasma from C57BL/6J mice was diluted 1:25 in PBS and analyzed by Western-blotting. Proteins were separated by 8% SDS-PAGE and transferred onto a nitrocellulose membrane using a Trans–Blot turbo transfer system. Membranes were incubated with rat anti–mouse primary antibody to VE-cadherin (0.25 μg/mL, Clone 11D4.1, reference 550548, BD Biosciences, Franklin Lakes, NJ, USA). After a rabbit anti-rat secondary antibody (1:2000) incubation (reference ab6734, Abcam, Cambridge, United Kingdom), membranes were revealed by chemiluminescence using a ChemiDoc XRS+ imager system. Nitrocellulose membranes, the transfer system, enhanced chemiluminescence detection reagents and the detection system were from Bio-Rad Laboratories (Marnes-la-Coquette, France). Bands were quantified by densitometry by using the public domain ImageJ software ). We also quantified sVE in 12 plasma samples from a pilot experiment from 6 N-exposed and 6 IH-exposed mice treated with the vehicle only (vehicle is PBS).

### 4.11. Immunofluorescence 

Cross-sections (thickness: 10 µm) of the cryopreserved thoracic aortas from C57BL/6J mice were used in immunofluorescence microscopy experiments. Cryosections were fixed in 4% paraformaldehyde for 15 min at room temperature. Non-specific binding was blocked with 10% goat serum in PBS. Sections were incubated overnight at 4 °C with a rat anti–mouse VE-cadherin primary antibody diluted at 1:50 (Clone 11D4.1 recognizing the extracellular domain of VE-cadherin, reference 550548, BD Biosciences, Franklin Lakes, NJ, USA). The slides were then incubated for 1-h with a secondary Alexa Fluor 488 anti-rat antibody diluted at 1:500 (references A-11006, Thermo Scientific, Waltham, MA, USA). Antibodies were diluted in PBS including 5% goat serum. Cell nuclei were stained with DAPI. One section from each aorta was observed by using a fluorescence microscope (Axio Imager, Zeiss, Oberkocheņ, Germany) and fluorescence was quantified using the software ImageJ after subtracting the autofluorescence of elastin and the background. 

### 4.12. Intima-Media Thickness (IMT) and Elastic Fiber Network Analysis

Cross-sections (thickness: 10 µm) of the cryopreserved thoracic aortas from C57BL/6J mice were fixed in 4% paraformaldehyde for 15 min at room temperature, then stained with hematoxylin-eosin or Weigert solution to assess IMT or elastic fiber defects, respectively. The sections were examined by microscopy (Axioscan, Zeiss, Oberkocheņ, Germany) and analyzed with the ZEN software version 3.1 (Zeiss). For each animal, four non-contiguous sections separated by about 100 µm were blindly analyzed. For IMT, a mean value was obtained by averaging 32 measurements (eight different areas per section). For the assessment of the level of elastic fiber alterations, the number of interruptions of the circumferential elastic lamellae, the number of elastic lamellae and the thickness of the elastic lamellae (made of the arterial wall elastic fibers) were measured in the entire aorta wall for each cross-section, a mean value was obtained by averaging three to four measurements.

### 4.13. Atherosclerotic Lesion Size

Whole aortas and aortic roots were harvested from ApoE-/- mice. Whole aortas were fixed in 2% paraformaldehyde for 24 h at 4 °C, then transferred in PBS and saved at 4 °C until staining. Cross-sections (thickness: 10 µm) of the cryopreserved aortic roots were fixed in 4% paraformaldehyde for 15 min at room temperature. Atherosclerotic lesions of the whole aortas and aortic roots were analyzed by Oil red-O (ORO) staining, which marks lipid depositions. Whole aortas were cut lengthwise and fixed by pins on a black support. For each aortic root, we observed five non-contiguous sections (thickness: 10 µm) separated by 100 μm using an Axioscan microscope (Zeiss, Oberkocheņ, Germany). Lipid deposition sizes were blindly measured in aortas and in aortic roots by using the software ImageJ (Fiji) and expressed as a ratio of the atherosclerotic lesion area (=ORO-stained area) to the total area of aortas or aortic roots in a given section. For each aortic root, a mean value was obtained by averaging the values obtained from at least four measurements.

### 4.14. Plasma Total-Cholesterol and LDL-Cholesterol Dosage

Total cholesterol and LDL-cholesterol in ApoE-/- mouse plasmas were analyzed by using an enzymatic colorimetric method on a chemistry analyzer (Atellica Solution, Siemens, Munich, Germany). 

### 4.15. Arterial Blood Pressure Recording

Systolic blood pressure (SBP), diastolic blood pressure (DBP) and mean arterial pressure (MAP) were measured by tail-cuff plethysmography in conscious ApoE-/- mice after 8 weeks of exposure to IH or N, by using the recording system CODA (Kent Scientific, Torrington, CT, USA). Mice were acclimated to the CODA restraining system and cuff inflation twice daily for three consecutive days before the effective recordings. For each mouse, the mean values of SBP, DBP and MAP were calculated by averaging the six successive measurements obtained during the effective recording.

### 4.16. Statistical Analysis

Statistical comparisons between two groups were performed by using a *t*-test or Mann–Whitney test, while comparisons of several groups were performed by using a three-way ANOVA, two-way ANOVA or Kruskal-Wallis test followed by the post hoc Fisher’s LSD test, using GraphPad Prism 8.0.1 software (GraphPad Software Inc., San Diego, CA, USA) or Statistica software (TIBCO Software, Palo Alto, CA, USA). The choice of parametric (*t*-test, ANOVA) or non-parametric (Mann-Whitney, Kruskal-Wallis) tests depended on the normality of the distribution of values and equality of variances. Values are reported as mean ± SEM or median + interquartile range (IQR), depending on the normality or not of their distribution. *p* ≤ 0.05 was considered as statistically significant.

## 5. Conclusions

We demonstrated that the inhibition of VE-cadherin cleavage pathways by the three inhibitors used was associated with the prevention of IH-induced vascular remodeling, elastic fiber alteration atherosclerosis and hypertension in vivo, as well as endothelial permeability to LDL and monocytes in vitro. These findings provide new insights into the IH-induced arterial dysfunctions and suggest a potential of VE-cadherin targeting for new anti-atherogenic, anti-hypertensive and anti-arterial stiffening therapeutic strategies to reduce cardiovascular complications associated to OSAS.

## Data Availability

Data are not publicly archived. Data analyzed and generated during the study are available from the corresponding authors O.H. and A.B.M. on reasonable request.

## Supplementary Materials

The following supporting information can be downloaded at: https://www.mdpi.com/article/10.3390/ijms23137012/s1.
