# Supplementary material for: Inhibition of Vascular Endothelial Cadherin Cleavage Prevents Elastic Fiber Alterations and Atherosclerosis Induced by Intermittent Hypoxia in the Mouse Aorta
Source: Int J Mol Sci. 2022 Jun 24;23(13):7012. doi: 10.3390/ijms23137012 (PMC9266969; doi:10.3390/ijms23137012)
Supplement: Supplementary file 1 [file ijms-23-07012-s001.zip › ijms-1740910-supplementary.pdf]

Supplementary Materials:

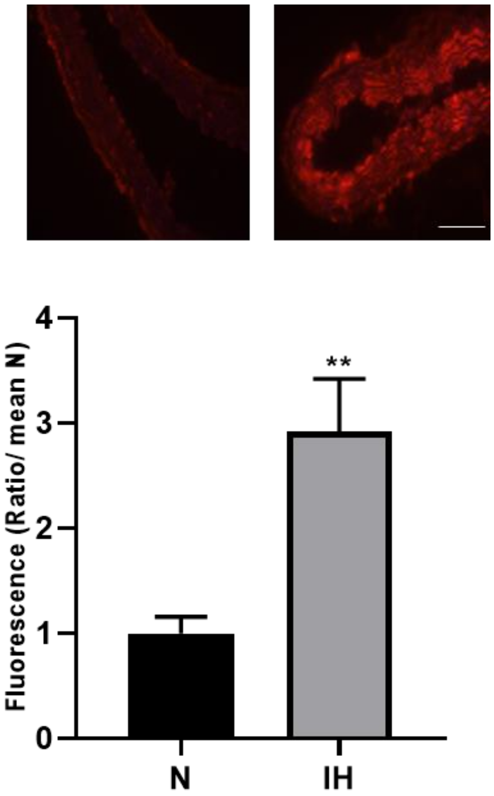

Supplementary data S1. The effect of IH was checked by measuring HIF-1 expression by immunofluorescence on aorta cryosections. Intermittent hypoxia activates HIF-1 in the aortic wall of C57BL/6J mice after 14 days of exposure to IH (N vs IH, p=0.006, Welch t-test, n=8). Values are mean + SEM. Crysections of 10um were fixed in 4% paraformaldehyde for 15 minutes at room temperature. Cryosections were incubated overnignt with a rabbit anti-mouse HIF1α diluted at 1:500 (Reference Ab2185, Abcam, Cambridge, United Kingdom), then with Alexa Fluor 546 anti-rabbit secondary antibody diluted at 1:500 (reference A-11035, Thermo Scientific, Waltham, USA). Scale bar, 50µm. ** p≤ 0.0.

Administration route	group	n	Weight day0	p-value	Weight day14	p-value	Hematocrit day14	p-value
Gavage	N	8	26,48±1,06	0,989	27,72± 1,03	<0,001	42,5±2	0,001
	IH	7	26± 1,11		25,45±1,01		48,85±3,07	
	N+Pazopanib	7	26,78±1,96	0,985	27,3±0,86	<0,001	44,42±2,99	0,03
	IH + Pazopanib	8	26,26±2,09		23,96±1,78		48,37±2,38	
	N+Saracatinib	6	26,75±0,94	0,878	27,61±0,43	<0,001	45,83±2,64	0,008
	IH+Saracatinib	5	27,78±1,57		24,72±1,27		52,5±1,29	
IP	N	8	25,07±2,13	0,992	26,41±1,9	0,004	44,5±1,19	0,006
	IH	8	25,3±1,03		23,83±1,03		49±4,14	
	N+ACF	7	24,48±1,72	0,788	25,84±0,98	0,001	45,28±1,25	0,002
	IH+ACF	8	25,28±1,67		22,82±1,29		51,37±2,55	

Supplementary data S2: Weight and hematocrit of C57BL/6J mice used for the vascular remodeling experiment.

Administration route	group	n	Weight day0	p-value	Weight day56	p-value	Hematocrit	p-value
Gavage	N	6	24,65±1,55	0,742	25,4 ±1,46	0,135	45±0,81	0,04
	IH	5	24,12±1,72		22,7±1		48,75±1,5	
	N+Pazopanib	6	26,38 ± 1,36	0,821	25,95 ±1,03	>0,999	44,83±2,13	0,05
	IH+Pazopanib	6	26,63 ± 1,4		25,11±1,7		48,83±3,25	
	N + Saracatinib	6	24,93 ± 0,64	0,47	24,61±0,47	>0,999	45,2±0,83	0,003
	IH + Saracatinib	6	25,51 ± 1,21		23,13±0,73		50,66±2,33	
IP	N	6	24,95±2,28	0,956	26,41±2,52	>0,999	47,50±2,88	0,73
	IH	5	24,56±1,2		24,38±0,98		48,25±2,21	
	N+ACF	7	24,58 ± 1,33	0,337	24,98 ± 1,79	0,363	45,14 ±2,96	0,11
	IH + ACF	8	23,62 ± 1,58		23,2±1,06		47,37 ± 2,82	

Supplementary data S3. Weight and hematocrit of ApoE-/- mice used for the atherosclerotic lesion experiments.

Administration route	group	n	Mean ± SD (mmol/L)	p-value
Gavage	N	6	10,62 ± 1,77	0,22
	IH	3	9,03 ± 0,65	
	N+Pazopanib	5	12,49 ± 1,71	0,36
	IH+Pazopanib	6	11,36 ± 0,99	
	N + Saracatinib	6	11,72 ± 1,89	0,22
	IH + Saracatinib	6	9,91 ± 3,16	
IP	N	6	9,02 ± 2,22	0,45
	IH	4	9,97 ± 3,61	
	N+ACF	6	10,10 ± 1,46	0,33
	IH + ACF	8	11,11 ± 2,86	

Supplementary data S4. Total cholesterol in ApoE-/- mice (n=3 to 5 in some groups, because of a lack of plasma to perform the dosage, due to technical issues).

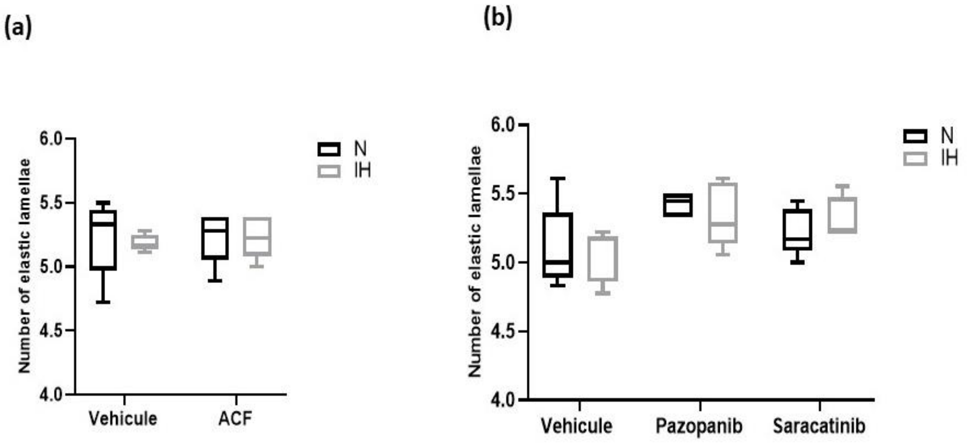

Supplementary data S5. **In aortic cross-sections, the number of concentric elastic lamellae was counted. IH and the three inhibitors (acriflavin, saracatinib and pazopanib) had no effect on the number of elastic lamellae in the aorta of C57BL/6J mice.** (a) Inhibiting HIF-1 (by ACF) had no effect on the number of elastic lamellae in the aorta of C57BL/6J mice (Kruskal-wallis test, p> 0.05, n=5). (b) Inhibiting VEGF receptor tyrosine kinases (by pazopanib) or src-kinases (by saracatinib) had no effect on the number of elastic lamellae in the aorta of C57BL/6J mice (Kruskal-wallis test, p> 0.05, n=5). N: normoxia, IH: intermittent hypoxia. Mice were exposed to N or IH for 14 days. Values are median + Interquartile range.
